# Prevalence and Risk Factors for Cannabis Use Among Tunisian Nursing Students

**DOI:** 10.1192/j.eurpsy.2026.10905

**Published:** 2026-07-31

**Authors:** A. Sdiri, I. Betbout, D. Charfi, Z. Ben Slimene, M. Ben Mbarek, N. Faouel, A. Ben Haouala, F. Zaafrane, A. Mhalla

**Affiliations:** 1 Psychiatry Research Laboratory LR05ES10 (Vulnerability To Psychoses), Faculty Of Medicine Of Monastir, University Of Monastir; 2 Psychiatry, Fattouma Bourguiba university hospital, Monastir; 3 UPSAT, Sousse, Tunisia

## Abstract

**Introduction:**

Cannabis use among young adults has become a growing public health concern in recent years. This age group is particularly susceptible due to several factors contributing to experimentation and regular use. As perceptions of cannabis risk decline and accessibility rises, understanding the prevalence and underlying risk factors is essential for informing effective prevention strategies and support interventions tailored to this population.

**Objectives:**

To assess the prevalence and risk factors associated with cannabis use among nursing students at a private institute in Tunisia.

**Methods:**

A cross-sectional analytical study was conducted involving 300 nursing students at a private higher education institute in Tunisia. Data were collected using a questionnaire, and cannabis dependence was assessed using CAST Test.

**Results:**

The study included 300 nursing students, predominantly female (63%), with most participants aged 20–21 years (49%). All were single, and the majority (96%) had not repeated an academic year. Over half (54.3%) lived with their families. Among the students surveyed, 33.7% reported having used at least one addictive substance, while 66.3% had never used any. Cannabis use was reported by 14% of students. Among cannabis users, 57.1% showed signs of probable dependence, while 42.9% reported occasional or low use. The average cannabis use score was 7.14, suggesting a trend toward frequent use and a higher risk of dependence. Cannabis use was not significantly associated with age (p = 0.135), but significant differences were found by sex (p < 0.001), with higher use among males (10%) than females (4%). Academic level was also significant (p = 0.002), with decreasing prevalence from first (8%) to third year (3.3%). Repeating a year was associated with use (p = 0.003), although proportions were similar (2%) between repeaters and non-repeaters. Living situation showed no significant effect (p = 0.133), but students living away from family reported slightly higher use (8% vs. 6%). Multivariate analysis confirmed sex as a key factor, with males more likely to use cannabis (OR = 0.215, 95% CI \[0.099–0.466]). Academic level also remained significant, with higher use in earlier years. The effect of sex was further confirmed in iteration 4 (OR = 0.175, 95% CI \[0.084–0.364]). Repeating a year showed a potential association (OR = 3.528, 95% CI \[0.940–13.243]), though not consistently significant.

**Conclusions:**

Cannabis use among students is relatively common, with a notable proportion showing signs of dependence. These findings highlight the need for targeted prevention strategies focusing on younger male students.

**Disclosure of Interest:**

None Declared

